# Blood Pressure Monitoring and Perinatal Outcomes in Normotensive Women with Gestational Diabetes Mellitus

**DOI:** 10.3390/jcm11051435

**Published:** 2022-03-05

**Authors:** Almudena Lara-Barea, Begoña Sánchez-Lechuga, Álvaro Vidal-Suárez, Ana I. Arroba, Fernando Bugatto, Cristina López-Tinoco

**Affiliations:** 1 Endocrinology and Nutrition Department, Hospital Universitario Puerta del Mar, 11009 Cadiz, Spain; almlarbar@gmail.com (A.L.-B.); bsanchezle@gmail.com (B.S.-L.); alvarovidal1992@gmail.com (Á.V.-S.); anaarroba@gmail.com (A.I.A.); 2 Biomedical Research and Innovation Institute of Cádiz (INiBICA), Hospital Universitario Puerta del Mar, 11009 Cadiz, Spain; fgbugatto@yahoo.com; 3 Obstetric and Gynecology Department, Puerta del Mar Hospital, 11009 Cadiz, Spain; 4 Area of Obstetrics and Gynaecology, Department of Child and Mother Health and Radiology, Medical School, Cadiz University (UCA), 11003 Cadiz, Spain; 5 Department of Medicine, Cadiz University (UCA), 11003 Cadiz, Spain

**Keywords:** gestational diabetes mellitus, ambulatory blood pressure monitoring, hypertensive disorders of pregnancy, perinatal outcomes

## Abstract

Alterations in ambulatory blood pressure detected by monitoring (ABPM) have been associated with perinatal complications in hypertensive pregnant women. Aim: To establish the relationships between the blood pressure (BP) profiles detected by ABPM and adverse perinatal outcomes in normotensive women with gestational diabetes mellitus (GDM). Methods: A prospective study of normotensive women in whom 24 h ABPM was performed at 28–32 weeks of pregnancy. The obstetric and perinatal outcomes were evaluated. Results: Two hundred patients were included. Thirty-seven women with GDM and obesity had significantly higher mean systolic BP (SBP) and nocturnal SBP and diastolic BP (DBP) compared to women with only GDM (*n* = 86). Nocturnal SBP (OR = 1.077; *p* = 0.015) and obesity (OR = 1.131; *p* = 0.035) were risk factors for the development of hypertensive disorders of pregnancy (HDPs). Mothers of newborns with neonatal complications (*n* = 27) had higher nocturnal SBP (103.8 vs. 100 mmHg; *p* = 0.047) and DBP (62.7 vs. 59.4; *p* = 0.016). Women who delivered preterm (*n* = 10) had higher BP and a non-dipper pattern (*p* = 0.005). Conclusions: Nocturnal SBP was a predictor of HDPs in normotensive women with obesity or GDM. Alterations in ABPM in these patients were associated with poor obstetric and perinatal outcomes.

## 1. Introduction

Hypertensive disorders of pregnancy (HDPs) imply an increase in maternal and neonatal morbidity and mortality as well as an increased risk of obstetric and perinatal complications [1,2,3]. HDPs include [3] gestational hypertension, defined as hypertension (systolic blood pressure (SBP) ≥ 140 mmHg and/or diastolic blood pressure (DBP) ≥ 90 mmHg) after the 20th week of gestation in women who were normotensive at baseline, and whose BP return to normal by 12 weeks after delivery; preeclampsia, the new onset of hypertension after the 20th week of gestation and develops proteinuria or end-organ dysfunction; chronic hypertension, defined as documented prepregnancy hypertension, use of antihypertensive medication before pregnancy or hypertension before 20 weeks of gestation and persisting 12 weeks after delivery; and preeclampsia over chronic hypertension, when preeclampsia appears in women with pre-existing chronic hypertension. These disorders affect 5–10% of pregnancies worldwide [4,5], and the presence of some comorbidities, such as gestational diabetes mellitus (GDM), can increase the risk of developing HDPs [6]. Furthermore, the prevalence of GDM has been increasing worldwide; this is related to the current obesity epidemic [7]. Therefore, it is necessary to design specific models that allow us to detect the development of HDPs in patients with GDM early in order to start preventive strategies in these women who are at increased risk.

Although isolated office blood pressure (BP) measurement remains the most commonly used method to detect hypertension in pregnancy in clinical practice, ambulatory blood pressure monitoring (ABPM) provides more reliable records and informs clinicians about BP variability over a 24 h period and the circadian rhythm [8]. In a healthy population, nocturnal BP physiologically falls by 10–20% compared to daytime BP values, which is known as a dipper pattern. The absence of this nocturnal BP fall (known as a non-dipper pattern) [9] and nocturnal hypertension have been associated with the development of HDPs in pregnant women [10,11,12]. These observations have led some authors, such as Salazar et al. [12,13], to recommend using ABPM in high-risk pregnancies (including women with pregestational diabetes) to detect early BP profile alterations in patients who subsequently develop HDPs. In pregnant women with GDM, there is insufficient evidence, but in a previously published study, we reported that high nocturnal SBP levels increase the risk of developing HDPs in pregnant women with GDM [14].

Previous studies have described an association between BP profile alterations in hypertensive pregnant women and obstetric and perinatal complications, such as preterm delivery, lower birth weight, and fetal growth restriction (FGR) [15,16,17]. However, few studies have related maternal and neonatal outcomes to BP variability in normotensive pregnant women [18,19].

The aim of the present study was to analyze the relationships between BP profiles (detected by ABPM) in normotensive pregnant women and risk factors for HDPs such as GDM and adverse obstetric and perinatal outcomes.

## 2. Materials and Methods

### 2.1. Study Design and Study Population 

We conducted a prospective observational study of 255 normotensive pregnant women attending a joint Endocrinology and Obstetrics clinic of the Puerta del Mar University Hospital (Cadiz, Spain), who were selected consecutively between August 2014 and December 2018. Women with GDM were divided according to their pregestational BMI (with or without obesity) and compared to non-diabetic women with normal weight (control group) and women with only obesity (without GDM). Only women with singleton pregnancies who delivered at the Puerta del Mar University Hospital were included. The following exclusion criteria were applied: women with pre-existing chronic hypertension or taking antihypertensive drugs at the time of recruitment, with a diagnosis of placental insufficiency, prepregnancy diabetes, morbid obesity (body mass index (BMI) > 40 kg/m^2^), smoking, and underlying chronic or acute systemic disease. The study protocol was approved by the Ethics Committee of the hospital (code number 1507-N-16) and conformed to the principles of the Declaration of Helsinki. Informed consent was obtained from all the participants.

Pregnant women at high risk of preeclampsia were advised to take 100 mg of aspirin daily from 12 weeks until 37 weeks, according to our hospital protocol based on NICE guidelines [20]. ASA prophylaxis was indicated in women at high risk with any of the following high-risk factors: hypertensive disease during a previous pregnancy, chronic kidney disease, autoimmune disease such as systemic lupus erythematosus or antiphospholipid syndrome, type 1 or type 2 diabetes, and chronic hypertension. ASA prophylaxis was also indicated in pregnant women with more than one moderate risk factor for preeclampsia: first pregnancy, age of 40 years or older, pregnancy interval of more than 10 years, BMI ≥ 35 kg/m^2^ at first visit, family history of preeclampsia, and multi-fetal pregnancy. Obesity was designated as having a prepregnancy BMI ≥ 30 Kg/m^2^. GDM was defined according to the criteria of the National Diabetes Data Group [21], and women with GDM were initially managed with diet. The glycemic targets during pregnancy were fasting glucose < 95 mg/dL and postprandial glucose at 1 h < 140 mg/dL. If blood glucose targets were not achieved with dieting in two weeks, insulin therapy was initiated. None of the women received oral hypoglycemic drugs.

Between 28 and 32 weeks of pregnancy, ABPM was performed on the nondominant arm during a 24 h period using a Spacelabs 90207 monitor (Spacelabs, Redmond, WA, USA). Measurements were scheduled every 20 min during the day and every 30 min at night. Patients were instructed to maintain normal daily activities, and sleep time was defined as the period between going to bed and rising the next morning. Only ABPMs with at least 66% successful measurements and at least one record per hour were considered valid. The following ABPM circadian patterns were established: the dipper pattern (BP decrease between 10–20% in the nocturnal period compared to the daytime period) and the non-dipper pattern (BP decrease < 10% in the nocturnal period compared to the daytime period).

On the day of the insertion of the ABPM device, fasting blood samples were collected at that moment for biochemical analysis, and maternal clinical data, method of conception, and obstetric history (including parity, previous history of GDM or macrosomia, history of gestational hypertension and/or preeclampsia) were obtained. Office BP was measured with an automated BP monitor (Omron HEM-7200-E (Kyoto, Japan)) in a sitting position twice on the same day and before ABPM.

The following obstetric and perinatal data were recorded after delivery: gestational age at delivery (gestational age was determined by first trimester crown–rump length measurements and was corrected in the case of a discrepancy of ±3 days with the last menstrual period), type of delivery, delivery route (eutocic, instrumental extraction, or cesarean section) delivery complications, birthweight, and the Apgar score. We documented complications of pregnancy such as HDPs, gestational hypertension (BP > 140/90 mmHg after the 20th week of gestation and return to normal BP levels in the postpartum period), preeclampsia (BP > 140/90 mmHg associated with proteinuria >300 mg/24 h or end-organ dysfunction), miscarriage, and intrauterine fetal death. Preterm delivery was considered as a delivery that occurred before 37 gestational weeks. FGR was defined as a birthweight less than the 5th percentile on a customized pediatric curve, small for gestational age (SGA) was designated as a birthweight below the 10th percentile, and large for gestational age (LGA) was when birthweight was greater than the 90th percentile for gestational age. Neonatal composite adverse outcomes were defined by the presence of hypoglycemia, hyperbilirubinemia, congenital malformations, or admission to the neonatal intensive care unit. 

### 2.2. Statistical Analysis

Data were processed and analyzed using IBM SPSS version 24.0 software for MS Windows. Descriptive statistics of the variables measured are presented as the mean and standard deviation (SD) for quantitative variables, and frequencies and percentages for the qualitative variables. We used the Shapiro–Wilk test to monitor the normality of the distributions. The X2 test (or Fisher’s exact test, as required) was used to compare qualitative variables, which does not allow us to specify the statistical significance between the four groups. The magnitude of association was calculated using the odds ratio (OR) with the precision described by the 95% confidence interval (95% CI).

Comparisons between quantitative variables and groups were performed with the Student’s t-test and one-way analysis of variance (ANOVA) for the parametric variables. Bonferroni post hoc tests were used to identify significant differences between specific groups in case of a significant F value from an ANOVA. Correlations among variables were evaluated using Pearson’s correlation test.

A multivariate analysis was performed using non-conditional logistic regression. The stepwise technique was used to select the independent variables introduced into the model. The goodness of fit of the final model was assessed using the Hosmer–Lemeshow test. *p* values less than 0.05 were considered statistically significant.

## 3. Results

**Figure 1:**
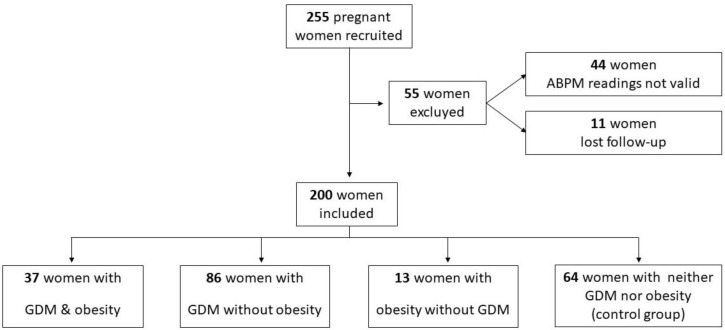
Study flow chart: algorithm for the identification and classification of eligible women. ABPM: ambulatory blood pressure monitoring; and GDM: gestational diabetes mellitus.

Two hundred and fifty-five pregnant women were recruited, and fifty-five women were excluded: 11 who gave birth elsewhere and 44 with ABPM readings that were not valid. We observed no significant difference in maternal age at enrollment, maternal pre-pregnancy BMI or gestational age between women who had valid ABPM data and completed the study when compared with the 55 women lost to follow-up. The remaining 200 women were divided into four groups (Figure 1): Group 1 (*n* = 37) were women with GDM and obesity, Group 2 (*n* = 86) were women with GDM without obesity, Group 3 (*n* = 13) were women with obesity without GDM, and Group 4 (*n* = 64) were women with neither obesity nor GDM (control group). 

Baseline clinical characteristics and laboratory features from each group are shown in Table 1. Maternal age was significantly higher in women with GDM. There was no significant difference in the family history of diabetes mellitus or hypertension, method of conception, history of polycystic ovarian syndrome, parity, or obstetric history (including antecedent of miscarriage, previous macrosomia, history of gestational hypertension, and/or preeclampsia) between groups.

Concerning the characteristics of pregnancy, office SBP and DBP were significantly greater in the group of women with GDM and obesity than in the group of women with only GDM, but there were no significant differences compared to women with obesity without GDM or between the other groups (Table 1). In relation to the laboratory variables, women with GDM and obesity showed higher levels of triglycerides and HbA1c. The rest of the measured variables were not significantly different between groups.

**Figure 2:**
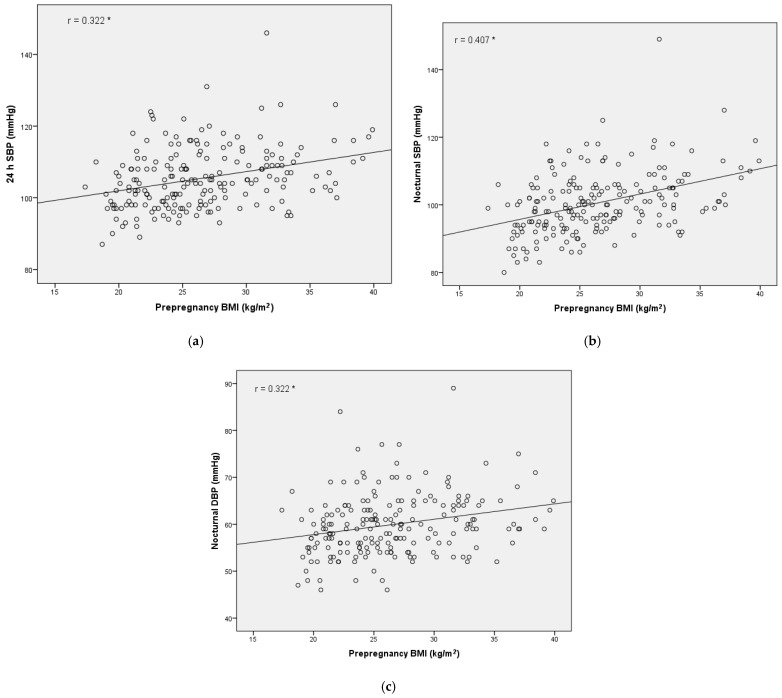
(**a**) Correlation between 24 h SBP and prepregnancy BMI. (**b**) Correlation between nocturnal SBP and prepregnancy BMI. (**c**) Correlation between nocturnal DBP and prepregnancy BMI. Linear correlation between ABPM parameters and prepregnancy BMI. Correlations were evaluated using Spearman’s correlation test r. * *p* < 0.01. ABPM: ambulatory blood pressure monitoring; SBP: systolic blood pressure; DBP: diastolic blood pressure; and BMI: body mass index.

With regard to ABPM parameters, there were some significant differences between groups (Table 1). The GDM with obesity group had significantly higher mean SBP, nocturnal SBP and DBP than women in the GDM without obesity group. We observed a significantly positive correlation between BMI and ABPM parameters (24 h SBP and nocturnal SBP and DBP), as shown in Figure 2.

Table 2 shows the pregnancy and obstetric and perinatal outcomes data. Among women with GDM (*n* = 123), the use of insulin therapy was higher in women with obesity (59.5% vs. 41.5%), but the total insulin doses were similar between the two groups (20.95 ± 17.06 vs. 19.92 ± 14.21 UI). All the other women with GDM were controlled with diet and lifestyle modifications. Prophylaxis with ASA was higher in patients with obesity, particularly those with added GDM.

Regarding the development of HDPs (including gestational hypertension and preeclampsia), the subgroup analysis showed no significant difference between the GDM and non-GDM groups (68.8% vs. 31.3%; *p* = 0.5). However, HDPs were higher in groups with obesity (56.3% vs. 43.8%; *p* = 0.005).

In the analysis of obstetric and perinatal outcomes (Table 2), delivery occurred earlier in diabetic groups, and the mean gestational age at delivery was lower in the GDM without obesity group. Weight gain was higher in non-GDM women, particularly those without obesity. No statistically significant differences were found in birthweight or neonatal composite adverse outcomes (hyperbilirubinemia, hypoglycemia, congenital malformations, or admission to the intensive care unit for any reason). Conversely, the rate of global delivery complications (including cesarean section during labor, instrumental delivery, and perineal tear) was significantly higher in GDM women with obesity than in the other groups.

To evaluate the relationship between the ABPM parameters and the development of HDPs, bivariate analysis was conducted, and the results are shown in Table 3. Pregnant women who developed HDPs had higher SBP (mean, daytime and nocturnal) and daytime DBP than normotensive women. Concerning the neonatal composite adverse outcomes, the nocturnal averages were significantly higher in women whose newborns had neonatal complications, as outlined in Table 3. We also identified a non-dipper pattern, and ABPM parameters of BP were significantly higher in women with preterm delivery than in mothers who had a term pregnancy. The remaining variables analyzed for obstetric and perinatal outcomes were not significantly different (results not shown).

Multivariate analysis used the development of HDPs as the independent variable and all the variables that were statistically significant in the univariate model were used as dependent variables, with some independent variables considered as having possible clinical significance. Table 4 summarizes the results of these analyses in the final model. The outcomes indicated nocturnal SBP (OR = 1.077) and BMI (OR = 1.131) as risk factors for the development of HDPs.

## 4. Discussion

In the present study, we found higher BP levels detected by ABPM in pregnant women who subsequently developed HDPs as well as adverse obstetric and perinatal outcomes. Our results suggest that ABPM could have clinical utility in the prediction of the risk for HDPs in pregnant women with comorbidities, such as GDM and prepregnancy obesity. To our knowledge, this is the largest sample size study on ABPM performed in women with GDM, and furthermore, we have included a group of patients with only obesity, without GDM, due to the close relationship between obesity and GDM [7] and the possibility that some of the results could be attributed to the presence of obesity, independently of the metabolic alterations present in GDM.

GDM and prepregnancy obesity are independent risk factors for maternal and neonatal complications [22,23,24,25,26]. In our cohort, the incidence of HDPs was 8% (*n* = 16), and no significant differences between groups were found with respect to the presence of GDM. However, obesity was observed to be an independent risk marker for developing HDPs, which is consistent with previous studies [23,27,28]. Moreover, when obesity is associated with GDM, the risk for HDPs increases compared to pregnant women with GDM without obesity, in agreement with other investigators [24,25,29,30]. On the other hand, while some authors found that the development of HDPs was even more frequent in pregnant women with obesity without GDM [24], others have not observed the same feature [30]. Either way, prepregnancy BMI seems to have a stronger influence than GDM on the development of HDPs [26].

Currently, there are no specific recommendations regarding the use of ABPM in pregnancy, except for the differentiation between true hypertension and the white-coat effect [31,32]. However, some authors recommend its usefulness in high-risk pregnancies [13]. In fact, Salazar et al. found a higher rate of masked hypertension (defined as a mean BP > 130/80 mmHg with normal office BP levels) and nocturnal hypertension in normotensive women with high-risk pregnancy, regarding these variables as predictors of the development of HDPs. In our cohort, we found higher 24 h, daytime, and nocturnal SBP and daytime DBP levels in pregnant women who developed HDPs. Furthermore, multivariate analysis identified nocturnal SBP as an independent risk factor for the development of HDPs, coinciding with the data published in relation to pregnant women with chronic hypertension or high-risk pregnancy [10,12]. In a cohort of normotensive pregnant women with GDM, we also observed that higher nocturnal SBP increased the risk of developing HDPs [14]. In addition, in this study, we found that patients in the GDM with obesity group had higher mean SBP, nocturnal SBP, and DBP than pregnant women with GDM without obesity. These data show that ABPM could have specific utility in this group with double risk, obesity and GDM. In this group, we also observed higher levels of triglycerides, in agreement with some authors [33,34], and postulated that this atherogenic profile could be related to endothelial dysfunction causing subclinical BP alterations, as has been described in subjects with type 1 diabetes mellitus [35]. Pregnant women with obesity and GDM also had higher HbA1c levels and received insulin therapy more frequently than GDM women without obesity, similar to findings reported by other investigators [24,29], but in our cohort, there was no difference in the total dose of insulin used between the two groups. Weight gain during pregnancy was significantly lower in the GDM groups than in the control group, which can be explained by specific dietary management and better compliance to recommendations in diabetic women. Nevertheless, inadequate weight gain has been related to the development of HDPs by other authors [29] as well as poor obstetric and perinatal outcomes [36].

In our study, the non-dipper pattern was more prevalent in the obesity groups, whereas women without obesity more frequently had a dipper circadian pattern. Although a significant relationship between the non-dipper pattern and the diagnosis of GDM in normotensive pregnant women has been described [14,37], these works do not analyze the influence of pre-pregnancy obesity as an independent factor in the ABPM pattern. In fact, there was a significant correlation between prepregnancy BMI and mean SBP and nocturnal SBP levels detected by ABPM (Figure 2) but not with blood glucose levels. These findings are consistent with the previous hypothesis reported [22,24,26] that considers prepregnancy obesity to have a more important role in the development of HDPs than GDM.

The incidence of HDPs in our patients was lower than expected, but it was higher in pregnant women with prepregnancy obesity even though ASA use was greater in this population. A possible explanation for these findings could be the use of low doses of ASA according to current recommendations in clinical practice guidelines [20,31] to reduce the risk of preeclampsia, preterm delivery, FGR, and miscarriage in pregnant women with chronic hypertension or risk factors for preeclampsia.

As described in the literature, gestational age at delivery was lower in pregnant women with GDM, probably due to the higher frequency of indications for induction in these patients compared to women with obesity without GDM. Most patients who had a preterm delivery showed a non-dipper pattern in ABPM, according to the data published by other authors, in pregnant women who develop gestational hypertension [16] and preeclampsia [38]. Furthermore, we observed significantly higher mean and nocturnal BP, both systolic and diastolic, and daytime SBP levels in these women compared to those with delivery at term, similar to that described previously in patients with preterm delivery associated with gestational hypertension [17].

The rate of delivery complications was higher in pregnant women with GDM and obesity, in agreement with other publications [24], but we did not find any differences with respect to birthweight, Apgar score, the rate of cesarean section, FGR, SGA, LGA or perinatal complications (composite variable) between the different groups. An association between the presence of FGR or SGA and circadian variation in BP in both normotensive and hypertensive pregnant women has been described in the literature [15,16,18,19]. In our study, BP values in ABPM were higher in women who had FGR, but these findings were not statistically significant. Nevertheless, we detected nocturnal SBP and DBP values that were significantly higher in pregnant women whose newborns had neonatal complications (hypoglycemia, hyperbilirubinemia, congenital malformations or admissions to the intensive care unit), as has been reported by some authors in women who subsequently developed gestational hypertension [17,39].

The principal limitation of our study was the small size of the obesity group without GDM (13 women), which could have influenced our ability to detect any differences from the other groups, and the inclusion of more patients could have provided greater precision for our results. However, the sample size was adequate to detect statistically significant and clinically relevant results.

Nevertheless, the most important strength is that this study is the largest sample size study evaluating the relationship between ABPM parameters and obstetric and perinatal outcomes in pregnant women with GDM considering obesity, and the inclusion of non-diabetic women both with and without obesity.

## 5. Conclusions

We concluded that normotensive pregnant women with GDM and obesity have a high risk of maternal and perinatal complications, with a presumably greater impact of obesity than GDM. Subclinical alterations in the BP profile in women with preterm delivery and neonatal complications have been described, and nocturnal SBP predicts the development of HDPs. Thus, ABPM may be useful for identifying women at higher risk of adverse obstetric and perinatal outcomes who could benefit from preventive actions. Studies with larger sample sizes would be necessary to confirm our results and allow us to find greater differences. 

## Figures and Tables

**Table 1:** Demographic, clinical, and laboratory variables, and ABPM parameters according to presence of GDM and/or obesity.

Variables	GDM and Obesity (*n* = 37)	GDM without Obesity (*n* = 86)	Obesity without GDM (*n* = 13)	Control (*n* = 64)	*p*-Value
Maternal age (y)	33.97 ± 3.956	34.96 ± 4.182	30.85 ± 5.414	33.36 ± 4.876	0.009 ^a^
Prepregnancy BMI (kg/m^2^)	33.81 ± 2.51	24.27 ± 3.02	32.97 ± 3.15	23.80 ± 2.79	<0.001 ^b^
Family history DM	19 (51.45%)	38 (44.2%)	6 (46.2%)	22 (34.4%)	0.4
Family history AHT	16 (43.2%)	38 (44.2%)	5 (38.5%)	23 (35.9%)	0.8
Parity					0.5
Nulliparous	16 (43.2%)	33 (38.4%)	7 (53.8%)	28 (43.8%)	
Multiparous	21 (56.8%)	53 (61.6%)	6 (46.2%)	36 (56.2%)	
Previous history of GDM	9 (24.3%)	21 (24.4%)	0	5 (7.8%)	0.01
Office SBP (mmHg)	114.7 ± 12.2	107.6 ± 15.6	116.2 ± 12.4	110.6 ± 11.3	0.02
Office DBP (mmHg)	70.9 ± 8.8	65.2 ± 8.6	67.6 ± 7.7	65.2 ± 8.5	0.005 ^c^
Total cholesterol (mmol/L)	6.13 ± 1.21	6.47 ± 1.22	6.27 ± 1.33	6.46 ± 0.99	0.5
LDL-cholesterol (mmol/L)	3.75 ± 1.48	3.77 ± 1.28	3.29 ± 1.15	3.46 ± 0.97	0.5
HDL-cholesterol (mmol/L)	1.91 ± 0.55	1.92 ± 0.43	1.89 ± 0.47	1.99 ± 0.54	0.9
Triglycerides (mmol/L)	2.55 ± 1.15	2.16 ± 0.72	2.46 ± 0.88	2.01 ± 0.7	0.012 ^d^
HbA1c (mmol/mol)	33.3 ± 2.62	30.4 ± 2.03	29.3 ± 2.03	29.2 ± 1.87	<0.001 ^e^
24 h SBP (mmHg)	109.19 ± 9.61	104.27 ± 8.46	108.15 ± 7.58	104.19 ± 7.45	0.009 ^f^
24 h DBP (mmHg)	66.73 ± 5.45	64.53 ± 6.66	64.38 ± 4.42	64.69 ± 5.45	0.3
Daytime SBP (mmHg)	110.89 ± 9.51	106.64 ± 9.15	109.15 ± 8.57	106.55 ± 8.04	0.06
Daytime DBP (mmHg)	68.54 ± 5.53	66.97 ± 6.90	65.77 ± 5.31	66.97 ± 5.86	0.46
Nocturnal SBP (mmHg)	105.84 ± 10.90	99.15 ± 8.68	105.31 ± 7.67	98.39 ± 7.60	<0.001 ^g^
Nocturnal DBP (mmHg)	62.70 ± 7.04	59.28 ± 7.08	60.38 ± 5.63	59.03 ± 5.49	0.034 ^h^
Non-dipper pattern	22 (59.5%)	42 (48.8%)	9 (69.2%)	20 (31.3%)	0.01

Values are expressed as means ± standard deviation. Categorical variables are given as the number of subjects (*n*) with the percentage in parenthesis. GDM = gestational diabetes mellitus; BMI = body mass index; DM = diabetes mellitus; AHT = Arterial hypertension; SBP = systolic blood pressure; DBP = diastolic blood pressure; LDL = low-density lipoprotein; HDL = high-density lipoprotein; and HbA1c = glycated hemoglobin. ^a^ GDM without obesity vs. Obesity without GDM *p* = 0.01. ^b^ GDM and obesity vs. GDM without obesity *p* < 0.001; GDM with obesity vs. control *p* < 0.001; Obesity without GDM vs. GDM without obesity *p* < 0.001; Obesity without GDM vs. control *p* < 0.001. ^c^ GDM and obesity vs. GDM without obesity *p* = 0.005; GDM and obesity vs. control *p* = 0.009. ^d^ GDM and obesity vs. control *p* = 0.01. ^e^ GDM and obesity vs. GDM without obesity *p* = 0.001; GDM and obesity vs. Obesity without GDM *p* = 0.007; GDM with obesity vs control *p* < 0.001. ^f^ GDM and obesity vs. GDM without obesity *p* = 0.018; GDM and obesity vs. control *p* = 0.02. ^g^ GDM and obesity vs. GDM without obesity *p* = 0.001; GDM and obesity vs. control *p* < 0.001. ^h^ GDM and obesity vs. GDM without obesity *p* = 0.05; GDM and obesity vs. control *p* = 0.04.

**Table 2:** Characteristics of pregnancy and obstetric and perinatal outcomes according to the presence of GDM and/or obesity.

Variables	GDM and Obesity (*n* = 37)	GDM without Obesity (*n* = 86)	Obesity without GDM (*n* = 13)	Control (*n* = 64)	*p*-Value
GDM treatment			-	-	0.05
Diet only	15 (40.5%)	50 (58.1%)			
Diet + insulin	22 (59.5%)	36 (41.9%)			
Total doses insulin (UI)	20.95 ± 17.06	19.92 ± 14.21			0.8
ASA prophylaxis	11 (29.7%)	7 (8.1%)	3 (23.1%)	3 (4.7%)	0.001
Development of HDPs	6 (16.2%)	5 (5.8%)	3 (23.1%)	2 (3.1%)	0.02
Preeclampsia	2 (5.4%)	2(2.3%)	0	1 (1.6%)	0.6
Gestational hypertension	6 (16.2%)	5 (5.8%)	3 (23.1%)	1 (1.6%)	0.007
Gestational age at delivery (week)	38.47 ± 1.30	36.16 ± 1.37	40.12 ± 0.92	39.54 ± 1.23	<0.001 ^a^
Preterm delivery < 37 week	4 (10.8%)	6 (7.0%)	0	0	0.06
Weight gain (kg)	7.30 ± 4.32	8.40 ± 4.17	9.15 ± 5.05	10.86 ± 4.29	0.001 ^b^
Instrumental delivery	10 (27%)	25 (29.1%)	1 (7.7%)	12 (18.8%)	0.1
Cesarean section	14 (37.8%)	19 (22.1%)	6 (46.2%)	16 (25%)	0.1
Childbirth complications	25 (67.6%)	39 (45.3%)	6 (46.2%)	22 (34.4%)	0.015
Birthweight (g)	3212 ± 615	3188 ± 465	3391 ± 421	3313 ± 475	0.3
FGR	3 (8.1%)	5 (5.8%)	1 (7.7%)	5 (7.8%)	0.9
SGA	7 (18.9%)	9 (10.5%)	2 (15.4%)	8 (12.5%)	0.6
LGA	5 (13.5%)	8 (9.3%)	0	8 (12.5%)	0.5
Neonatal adverse outcomes	8 (21.6%)	12 (14.2%)	2 (15.4%)	5 (7.8%)	0.3

Values are expressed as means ± standard deviation. Categorical variables are given as the number of subjects (*n*) with the percentage in parenthesis. GDM = gestational diabetes mellitus; ASA = acetylsalicylic acid; HDPs = hypertensive disorders of pregnancy; FGR = fetal growth restriction; SGA = small-for gestational age; and LGA = large-for-gestational age. ^a^ GDM and obesity vs. GDM without obesity *p* = 0.044; GDM and obesity vs. Obesity without GDM *p* = 0.001; GDM with obesity vs. control *p* = 0.001. ^b^ GDM and obesity vs. control *p* = 0.001; GDM without obesity vs. control *p* = 0.006.

**Table 3:** Bivariate analysis of the association between BP parameters, analyzed by ABPM, and develop of HDP, preterm delivery and the presence neonatal composite adverse outcomes.

Variables	Development of HDPs		Preterm Delivery		Neonatal Adverse Outcomes	
	Yes (*n* = 16)	No (*n* = 184)	Yes (*n* = 10)	No (*n* = 190)	Yes (*n* = 27)	No (*n* = 173)
24 h SBP (mmHg)	113.1 ± 13.5 *	104.7 ± 7.6 *	114.5 ± 14.5 *	104.9 ± 7.8 *	107.9 ± 11.1	105 ± 7.9
24 h DBP (mmHg)	68.8 ± 8.9	64.6 ± 5.5	72.1 ± 8.1 *	64.6 ± 5.6 *	67.0 ± 7.9	64.6 ± 5.5
Daytime SBP (mmHg)	115.6 ± 12.5 *	106.9 ± 8.2 *	115.9 ± 13.6	107.1 ± 8.4	109.7 ± 10.8	107.2 ± 8.5
Daytime DBP (mmHg)	71.1 ± 8.1 *	66.8 ± 5.9 *	73.6 ± 7.8 *	66.8 ± 5.9 *	68.9 ± 8.0	66.9 ± 5.9
Nocturnal SBP (mmHg)	107.8 ± 16.7 *	99.9 ± 7.9 *	112.0 ± 17.4 *	99.9 ± 8.1 *	103.8 ± 13.2 *	100 ± 8.3 *
Nocturnal DBP (mmHg)	62.9 ± 11.4	59.6 ± 5.9	68.9 ± 9.8 *	59.4 ± 6.1 *	62.7 ± 8.6 *	59.4 ± 6.1 *
Non-dipper pattern	7 (43.8%)	86 (46.7%)	9 (90.0%) *	84 (44.2%) *	15 (55.6%)	78 (45.1%)

Values are expressed as means ± standard deviation. Categorical variables are given as the number of subjects (*n*) with the percentage in parenthesis. * *p* < 0.05. HDPs = hypertensive disorders of pregnancy; SBP = systolic blood pressure; and DBP = diastolic blood pressure.

**Table 4:** Final model of the multivariable regression analysis for the risk prediction of HDP. B: Beta; Exp (B): beta exponent; 95% CI for Exp (B): 95% confidence interval for beta exponent. GDM = gestational diabetes mellitus; BMI = body mass index; and SBP = systolic blood pressure.

Variables	B	*p*-Value	Exp (B)	95% CI for Exp (B)
Nocturnal SBP	0.074	0.015	1.077	1.015–1.143
Prepregnancy BMI	0.123	0.035	1.131	1.009–1.268
Dipper pattern	0.990	0.112	2.691	0.793–9.130
GDM	0.077	0.902	1.080	0.316–3.685
Age	−0.033	0.601	0.967	0.854–1.096
